# Examining the relationships among physician implicit bias, language, and Hispanic patient satisfaction

**DOI:** 10.1371/journal.pone.0338699

**Published:** 2025-12-31

**Authors:** Katie Wolsiefer, Jeff Stone, Matthias R. Mehl, Gordon B. Moskowitz, Colleen K. Cagno, Colin A. Zestcott, Alma Tejeda Padron

**Affiliations:** 1 Department of Psychology, Appalachian State University, Boone, North Carolina, United States of America; 2 Department of Psychology, Northern Arizona University, Flagstaff, Arizona, United States of America; 3 Department of Psychology, University of Arizona, Tucson, Arizona, United States of America; 4 Department of Psychology, Lehigh University, Bethlehem, Pennsylvania, United States of America; 5 College of Medicine, University of Arizona, Tucson, Arizona, United States of America; 6 Department of Psychology, College of Saint Scholastica, Duluth, Minnesota, United States of America; Universitair Kinderziekenhuis Koningin Fabiola: Hopital Universitaire des Enfants Reine Fabiola, BELGIUM

## Abstract

**Objective:**

The present study examined links among physician implicit bias and physician word use and patient satisfaction during outpatient medical visits.

**Method:**

To test these relationships, we measured implicit anti-Hispanic bias of 53 internal and family medicine residents and audio recorded outpatient visits between these residents and 291 of their Hispanic-identifying patients between 2015 and 2017. After each visit, both patients and resident physicians completed surveys measuring their perceptions of the interactions (residents) or their satisfaction with their care (patients). Linguistic Inquiry and Word Count was used to quantify resident word use from each audio recording.

**Results:**

A quadratic relationship between physician implicit bias and patient satisfaction suggested that increased levels of implicit anti-Hispanic bias were related to lower levels of patient satisfaction, but only at high levels of physician bias. Exploratory analyses revealed that several language variables interacted with physician implicit bias to predict patient satisfaction suggesting that implicit bias may also be communicated by more moderately biased physicians in some contexts.

**Conclusions:**

These results add to a growing literature establishing links between physician implicit bias and patient care and suggest that future work should explore how context impacts the communication of physician bias to patients.

## Introduction

The individual biases held by medical providers may contribute to patient satisfaction following medical care, especially for patients from minority groups. Racial and ethnic health disparities are well documented in the United States [1–3] and there is substantial evidence that healthcare providers, like the general public, demonstrate a wide variety of implicit biases towards a wide range of marginalized groups [4,5]. Implicit biases refer to associations between a social category (e.g., Hispanic people) and a trait (e.g., “non-compliant”) or evaluation (e.g., “good”) that are considered more automatic than explicit, or self-reported, biases either because individuals are less aware that they hold these biases or because these biases inform behavior outside of conscious awareness [6]. Evidence suggests that providers associate individuals who are racial and ethnic minorities, people with obesity, members of the LGBTQIA+ community, people with disabilities, or people who have stigmatized health conditions with more negative concepts compared to individuals who are white, thin, straight, able bodied, and without stigmatizing health conditions [4,7–12]. The implicit prejudices and negative stereotypes that providers hold about patient groups may create barriers to providing equitable care.

Evidence tying provider biases to patient care varies based on the type of outcome considered. Most studies examining if provider implicit bias links directly to medical decision making have shown that, across a wide variety of health care providers (nurses, internal and family medicine physicians, surgeons, pain management specialists, etc.), the relationship between these biases and treatment recommendations is small and unreliable [13–20]. However, research examining the relationship between provider bias and patient perceptions of care suggests that physician bias may be more reliably linked with interpersonal aspects of patient care. Black patients whose doctors demonstrate greater anti-Black implicit prejudice report less positive attitudes toward their healthcare providers [21,22], less confidence in completing treatment recommendations, perceive fulfilling treatment recommendations as more difficult, and remember less about their encounter with more biased physicians [22]. Additionally, physicians who hold stronger implicit non-compliance stereotypes towards Black people have patients who feel less involved in their medical care and trust their physicians less [21]. One pilot study suggests that physician implicit bias correlates with broader aspects of patients’ well-being [23]. In a study examining the relationship between physician implicit bias and Black spinal cord injury patients, greater implicit racial bias was associated with higher rates of patient depression, lower life satisfaction, and less ability to maintain social relationships. Whereas racial concordance between physicians and patients can attenuate the relationship [21–23], these findings suggest physicians communicate biases to patients that are reflected in their perceptions of medical care and, potentially, to patient well-being. Nevertheless, an important limitation to the previous work is that it has focused largely on the relationship between provider bias and outcomes for Black patients. It is important examine if such patterns extend to other racial and ethnic patient groups, such as Hispanics, who are the largest ethnic minority group in the US [24].

Another potential limitation is that each of the above studies presents the relationship between physician implicit bias and patient perceptions of care as a simple bi-variate relationship. One study, drawing on aversive racism theory [25,26], examined the extent to which the effects of implicit bias on Black patient perceptions were moderated by explicit bias [27]. Overall, patients with more implicitly biased physicians were less likely to view themselves and their physician as a team. Additionally, physician explicit bias moderated the effect of implicit bias on patients’ overall feelings about their medical care. Consistent with aversive racism theory, Black patients had more negative perceptions of their care when physicians were high in implicit but low in explicit bias compared to any other combination of implicit and explicit bias. These findings suggest that implicit bias may not always operate directly on patient perceptions, but may be selectively communicated depending on other interpersonal factors, such as the language that providers use during a clinical interaction.

Although providers appear to communicate bias to patients non-verbally, relatively little is understood about whether or how physicians’ verbal behaviors factor into these interactions. Word choice is often intentional and can communicate meaning through the semantic content of words speakers decide to use. However, there is also evidence that word choice (e.g., pronouns, verbs, and prepositions) can communicate information about the speaker’s motivations, intentions, and their identity [28–30]. For example, participants asked to write an essay about a time they felt more powerful were more likely to speak about work-related topics [31]. Other studies find that using nouns compared to adjectives to describe others (e.g., “Jane is a liar” vs. “Jane was dishonest”) is associated with more essentialist beliefs [32], and that greater use of first person singular pronouns (e.g., “I”, “me”,”my”) is associated with greater attention to the self and stronger perceptions of narcissism [29,33]. Thus, physicians who implicitly associate Hispanic patients with negative traits and emotions may also view themselves as more powerful, hold more essentialist beliefs about their patients [34], or engage in more self-focused attention during interactions with Hispanic patients. To the extent that a physician’s subtle word choice unintentionally communicates these internal states during clinical interactions with Hispanic patients, they may negatively influence the patients perceptions of the care they receive.

Additionally, research suggests that context, intention, and longevity of relationship can all inform the ways in which language is interpreted [30,35–38]. For example, language style matching or the extent to which two individuals align in their use of function words is often associated with positive interpersonal outcomes such as greater liking and relationship satisfaction [30,37]. However, in the early stages of transactional relationships, greater language style matching is viewed as a marker of deception [38]. Thus, the same language patterns can be perceived differently depending on context. In a medical visit, physician non-verbal expressions of bias may create contexts in which word use is interpreted differently by their patients than when unbiased physicians use the same language.

To date, only three studies have examined the relationship between physician implicit bias and verbal behavior [21,39,40]. Greater physician implicit racial bias is associated with both how physicians use language [21,41] and with physician word choice [39,42]. Hagiwara and colleagues (2017) measured the implicit anti-Black prejudice of 18 non-Black primary care physicians and audio recorded their interactions with 117 Black patients. Physician word use was quantified using the Linguistic Inquiry and Word Count (LIWC) software [43]. They found that physicians with greater anti-Black implicit bias used more first-person plural pronouns and anxiety-related words compared to their less biased colleagues. The finding related to first-person plural pronouns may seem counterintuitive. Although first-person plural pronouns can be used to a shared group, it may also an indicator of interpersonal coldness in some contexts (Zimmerman et al., 2013). Similarly, Wolsiefer and colleagues (2023) examined physician implicit biases towards Hispanic patients in a larger sample including both Hispanic and non-Hispanic physicians (*N* = 59 residents, *N* = 291 patients). Their findings suggest that physicians with greater implicit anti-Hispanic bias used more interrogatives and work-related language, but fewer prepositions and words related to the future, relativity, and time (these effects did not differ by ethnicity of the physician). These differences in findings may result from sampling from different populations of physicians (e.g., residents vs. more established physicians, physicians in the U.S. Midwest vs. Southwest, etc.) or from a focus on biases towards two different target groups (e.g., Black vs. Hispanic patients). Notably, all of these studies examine whether physician word use is directly related to physician bias but did not examine how verbal behavior and physician bias predict patient perceptions of care.

The goal of the present study is to examine the relationship between physician implicit bias, verbal behavior and patient perceptions of their care by conducting a secondary analysis of an existing dataset of approximately 300 doctor-Hispanic patient clinical interactions [40]. The present analysis extends the findings of Wolsiefer et al. (2023) by examining the relationship between physician implicit bias and patient satisfaction, and how this relationship may vary as a function of the words that physicians with different levels of implicit bias use when communicating with Hispanic patients. A secondary analysis of this large-scale study has the potential to provide further insight into the correlates of physician bias with actual patient interactions.

## Method

### Transparency and openness

In this article we report details about how we determined sample size, exclusions, information about all measures. All measures, analysis code, and a de-identified version of the dataset can be found on the OSF website: https://osf.io/6pez7/?view_only=697757185ad945559c9c29d7b081dc65. These studies were not pre-registered and all analyses except those marked as exploratory were blinded ex post. An analysis of the linguistic markers of physician implicit bias has been published based on this study [40] but differs from the current analysis in that it focused solely on correlates between implicit bias and physician word use. In contrast, this paper examines the relationship between physician bias and patient satisfaction and language use as moderators of this relationship.

### Participants

This research was submitted for ethics review and approved by the University of Arizona Institutional Review board, protocol number 1406364892A010. Resident physicians (resident physicians are physicians who have graduated from medical school and are pursuing specialization) in Internal Medicine and Family Medicine at the University of Arizona and their patients were recruited for this study between March 15, 2015 and March 15, 2017. Power analysis [44] suggested that 50 resident physicians (seeing 5 patients each) were needed to achieve our desired level of statistical power for the primary analyses of the grant-funded study/parent project. To achieve this sample size, we recruited all willing resident physicians contacted over a 6-month period. Resident physicians were recruited by members of the research team and provided informed consent during continuing education sessions and during shifts at an outpatient medical clinic. All Hispanic patients were recruited by members of the research team and provided informed consent at the start of an outpatient medical visit. All residents and patients were informed that decisions to participate in this study (or not) would have no bearing on either their employment/training (residents) or medical care (patients). We analyzed the final sample outlined in Wolsiefer et al. (2023). After exclusions (described in Wolsiefer et al., 2023), the final sample consisted of 53 resident physicians (29 female, 24 male, 49% White, 28% Asian or Asian American, 11% Hispanic (we chose to use the term Hispanic rather than the NIH approved “Hispanic or Latino” in an effort to be more gender inclusive), all other races < 6%, M_age_ = 29.83, SD_age_ = 3.61).

The patient sample consisted of 291 (183 female, 100 male, 8 unreported gender, 95% Hispanic, all other races < 5%, M_age _= 46.36, SD_age_ = 16.49) participants. To aggregate across meeting with patients, each resident physician provided between 2 and 9 recordings (*M* = 5.62 patients, *SD* = 1.33).

### Procedure

After consenting, resident physicians completed online measures of implicit and explicit bias, their approach to intergroup interactions, demographics and medical training experiences. Approximately 2–4 weeks later we began recording outpatient visits.

Hispanic patients were recruited after arriving for outpatient office visits and being assigned to an exam room, prior to seeing a resident physician. Outpatient visits took place in family and internal medicine clinics and were not restricted to specific medical conditions or reason for visit. After obtaining patient consent, an audio recording device (TASCAM DR-100MKiii audio recorder) was used to record the visit with the patient and resident. After the clinic visit, the resident and the patient completed survey measures of their perceptions of the clinic visit in different areas of the clinic. In addition, the patients completed a demographic questionnaire that included questions about their age, gender, race, ethnicity, first language, years speaking English, SES, and parents’ country of origin.

### Measures

#### Resident-level measures.

**Implicit racial bias.** Resident physicians first completed two Implicit Association Tests (IAT) [45] to measure implicit prejudice (unpleasant versus pleasant associations to the group) and stereotyping (noncompliance associations to the group) of Hispanic patients. The order in which participants completed compatible and incompatible test blocks, as well as the order of the Prejudice IAT and Stereotype IAT, was counterbalanced.

After completing practice blocks to familiarize themselves with the stimuli, each IAT required participants to simultaneously sort Hispanic vs. White faces and words from two opposing attribute categories using only 2 response keys. For the prejudice IAT these words were “pleasant” vs. “unpleasant” words (e.g., ‘love’,’peace’, ‘loyal’ vs. ‘disaster’,’evil’,’rotten’). For the stereotype IAT, these words were “compliant” vs. “noncompliant” words (e.g., ‘cooperative’,’compliant’,’helpful’ vs. ‘apathetic’,’resistant’,’reluctant’). For half of all test trials, Hispanic faces and the positive attribute category (either “pleasant” or “compliant) shared a response key while White faces and the negative attribute category (either “unpleasant” or “noncompliant”) shared a response key. For the remaining test trials, the response mappings were flipped so that Hispanic shared a response key with the negative category and White shared a response key with the positive category. Response latencies for each correct response were recorded.

All response latencies for each participant were converted into a *d-*score [45], the typical metric used to quantify implicit associations measured by the IAT. The d-score is a standardized difference in response latencies on the test blocks where Hispanic shared a response key with positive words compared to test blocks where Hispanic shared a response key with negative words. Higher *d-*scores indicated greater implicit prejudice towards Hispanics or the stereotypic association of Hispanic with noncompliant. For details on the implementation of this measure, see Wolsiefer et al. (2023).

**Approaches to intergroup interactions.** Participants completed several other scales to measure motivational and emotional aspects of resident physicians’ approaches to interactions with members of other groups. First, they completed the Internal (IM) & External (EM) Motivation to Respond without Prejudice scale [46] ($\alpha_{\text{IM}}=\text{0}.\text{70}$, $\alpha_{\text{EM}}=\text{0}.\text{75}$) to examine the extent to which they feel internal and external pressure to appear non-biased. Next, they completed the perspective taking subscale of the Perspective Taking and Empathic Concern Scale [47] (1983; α=0.83). Participants completed items assessing the extent to which they believe prejudice is immutable [47] (α=0.88), a single item assessing receptiveness to feedback about racial bias, a measure of colorblind ideology (α=0.89) and self-reported how they would respond to difficult interracial interactions [48].

#### Patient-level measures.

**Language data.** For each patient visit, the audio recording was professionally transcribed (by Datagain, a HIPAA-compliant transcription and translation service; https://transcription.datagainservices.com/). Any Spanish language on the recordings was transcribed in Spanish and then translated verbatim into English. Finally, a trained bilingual research assistant reviewed the transcript and translation for accuracy. Resident language was separated from the transcript and processed using the Linguistic Inquiry and Word Count software (LIWC) [43]. LIWC is currently the best validated and most widely used dictionary-based text analysis tool in the social science [49]. LIWC analyzes text by comparing each word in a text document to an internal dictionary. LIWC calculates the percentage of total words in a given text that match a set of pre-determined grammatical or semantic category of target words (e.g., articles, prepositions, or work-related words). Table A in S1 Table has been reproduced from Wolsiefer et al. (2023) to provide a list of the LIWC language categories and examples. In addition to the language categories provided by LIWC, we also calculated a single language style matching (LSM) score for each outpatient interaction. This metric examines the extent to which physicians and their patients are matching the use of 9 categories of function words during the outpatient visit. This variable was calculated using an approach described in Ireland and Pennebaker (2010) [50]. Higher scores indicate that resident physician’s and patient’s use of function words was more similar during the visit. The LSM index has generally been used as a marker of relational rapport and “being in sync” [51].

**Patient satisfaction.** After their outpatient visits, patients used a 7-point Likert-type scale to rate their agreement with seven statements regarding their impressions of their doctor. Items included reflected self-appraisals (e.g., “This doctor likes me”) and ratings of their own opinion of the doctor (“I trust this doctor to look out for my best interests”). One item – “If there were a choice between treatments, how often do you believe your doctor would ask you to help make decisions?”—was excluded from analysis because the Likert-type response scale did not make sense given the wording of the item. Cronbach’s alpha for the remaining 6 items was very high (α = 0.97) so these items were averaged to form a single patient satisfaction score ranging from 1 (least satisfied) to 7 (most satisfied).

**Resident perceptions of visit.** After each outpatient visit, residents were asked to rate themselves during the clinic visit on 5 different dimensions: pleasant, harsh, rude, unlikeable, and cold. These ratings were made on a 5-point scale ranging from 1 (not at all) to 5 (extremely). Residents were also asked to take the patient’s perspective and rate their agreement with the statement “I like this doctor” on a 5-point scale ranging from 1 (strongly disagree) to 5 (strongly agree). These items were also highly internally consistent (*ɑ* = 0.84). The items were averaged to form an index of resident physician perceptions of the visit ranging from 1 (unfavorable perception of visit) to 5 (favorable perception of visit). We report the use of this measure for transparency, but this measure was not analyzed and will not be discussed further.

## Results

### Implicit bias and patient satisfaction

As reported in Wolsiefer et al. (2023), average scores for implicit anti-Hispanic stereotypes and prejudice were both positive and significantly greater than 0, indicating that resident physicians associated Hispanic individuals with both negative evaluations and non-compliance stereotypes (compared to White individuals), stereotypes: *M* = 0.26, *SD* = 40, t(52) = 4.73, *p* < .001, *Cohen’s D* = 0.65; prejudice *M* = 0.37, *SD* = 42, t(52) = 6.41, *p* < .001, *Cohen’s D* = 0.88. Scores on the measure of implicit prejudice and implicit stereotyping were highly correlated, *r*(51) = 0.64, *p* < 0.001, *95% CI* (0.44, 0.77), so we averaged them to create one overall implicit bias score for each resident physician [40].

Given the racial diversity of physicians in this sample, it was possible that physicians differed in implicit bias based on their own racial/ethnic identity. A previous analysis of these data did not find evidence of race differences in physician implicit bias (40). Additionally, we tested whether race moderated the linear or quadratic effects of implicit bias on patient satisfaction. There was no evidence that this was the case (race X IAT linear: *b* = −.35, *t*(283) = −0.94, *p* = 0.35, *95% CI* [−1.07, −.37]; race X IAT quadratic: *b* = 0.99 *t*(281) = 1.17, *p* = 0.24, *95% CI* [−0.64, 2.62]. Since physician bias did not differ as a function of race/ethnicity, we did not include physician race/ethnicity as a factor in subsequent analyses.

**Fig 1:**
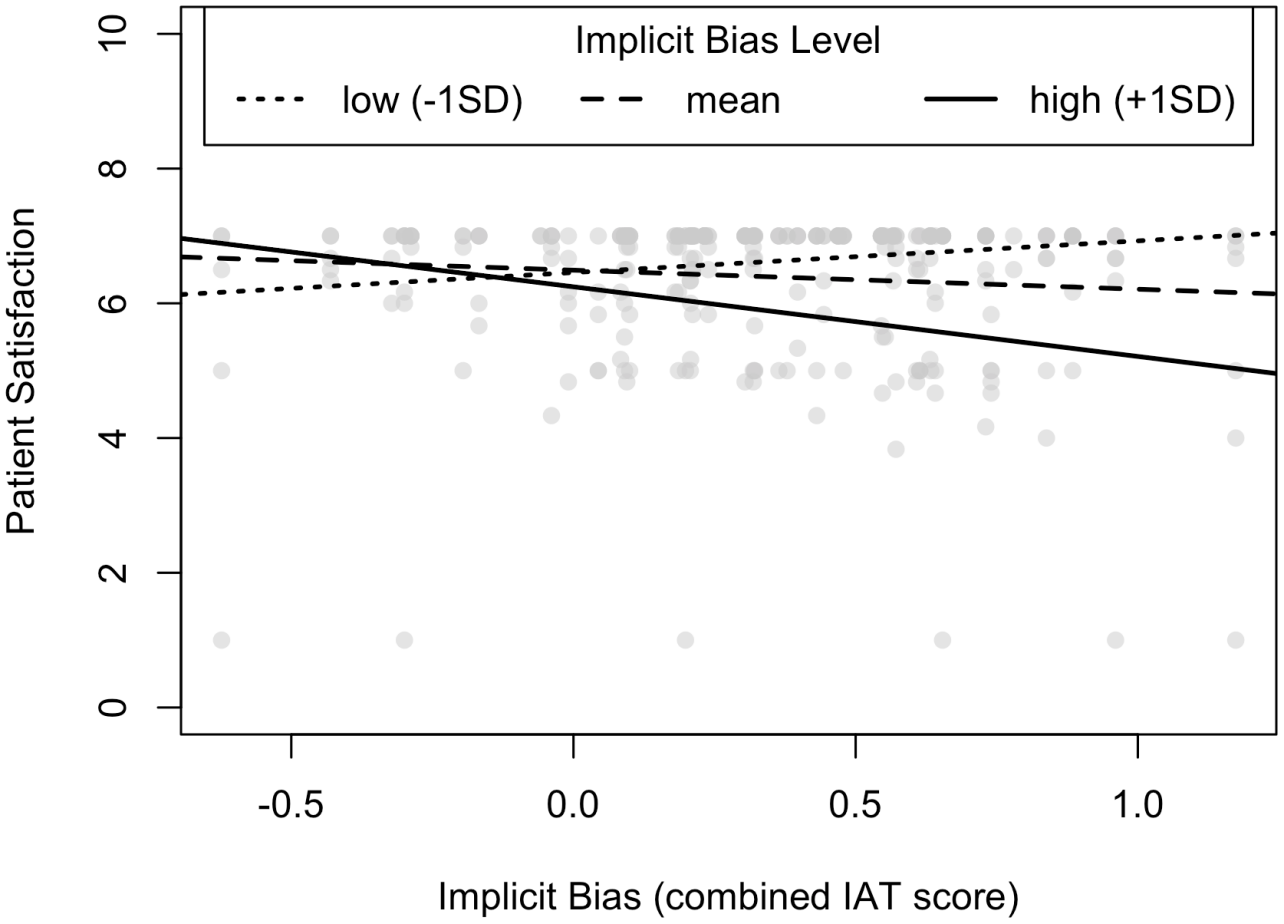
Quadratic relationship between physician implicit bias and patient satisfaction. *Note.* Figure represents relationship between physician implicit bias and patient satisfaction at −1 SD, mean and +1SD levels of bias.

To examine whether physician implicit bias was related to patient satisfaction, we estimated a linear mixed effects model. This model regressed patient satisfaction on physician implicit bias and estimated a random intercept to account for non-independence since patients were nested within residents. Unlike in previous research [21], there was no evidence of a linear relationship between resident bias and patient satisfaction, *b* = −0.22, *t*(285) = −1.23, *p* = 0.22, *95% CI* (−0.56, 0.13). However, we reasoned that it is possible that patients may only be sensitive to their physician’s implicit biases at relatively high levels, which would correspond to a non-linear effect. Thus, in a subsequent exploratory analysis, we tested a non-linear relationship between physician implicit bias and patient satisfaction and found a significant quadratic effect, *b* = −1.01, *t*(284) = −2.93, *p* = 0.004, *95% CI* (−1.69, −.34). This effect remained significant whether or not Hispanic identifying physicians were excluded from the analysis. To interpret this quadratic effect, we examined the simple effect of implicit bias on patient satisfaction at one standard deviation below the mean IAT score, the mean, and one standard deviation above the mean IAT score. High levels of physician implicit bias were significantly related to patient satisfaction, *b* = −1.04, t(284) = −3.15, *p* = 0.002, *95% CI* (−1.68, −0.39), $\hat{PSAT}$ = 6.25. However, at low and mean levels of implicit bias, there was no evidence of a relationship between implicit bias and patient satisfaction, low: *b* = 0.47, *t*(284) = 1.60, *p* = 0.11, *95% CI* [−0.10, 1.04], $\hat{PSAT}$ = 6.46; mean: *b* = −0.28, *t*(284) = −1.59, *p* = 0.11, *95% CI* [−0.63, 0.06], $\;\hat{PSAT}$ = 6.49. This suggests tha*t*, among physicians with higher levels of implicit bias, implicit bias was associated wi*t*h lower levels of patient satisfaction (see Fig 1). Whether physicians self-reported any implicit bias training in the past did not relate to patient satisfaction or moderate the effect of implicit bias (linear or quadratic effects) on patient satisfaction, all ps > 0.22.

### Resident language

To examine whether physician language played a role in whether physician implicit bias was communicated to patients, we conducted 3 additional analyses. For each of the 3 sets of models below, we began by examining language variables that previous literature has deemed relevant for physician-patient interactions, namely: 1^st^ person singular pronouns (e.g., “I”, “me”), anxiety words (e.g., “worried”, “fearful”, interrogatives (e.g., “how”, “who”), work-related language (e.g., “job”, “xerox”), prepositions (e.g., “to”, “with”), future words (e.g., “may”, “will”), relativity words (e.g., “area”,”bend”), time-related words (e.g., “end”,”until”), and language style matching. After running analyses on these variables, we conducted exploratory analyses using every variable provided by the LIWC program.

In the first analysis, we examined quadratic effects of physician implicit bias on the physician word use found in previous research. To test these relationships, we estimated a series of mixed effects models that regressed each of these LIWC language variables on the linear and quadratic effects of resident implicit bias and allowed the intercept to vary by resident. There was no evidence of a quadratic relationship between implicit bias and any of the LIWC language categories even before performing any corrections for multiple significance tests, all ps > .06. See Table B in S1 Table for a summary of these quadratic effects.

Second, we examined the relationship between resident physician language and patient satisfaction by estimating linear mixed effects models that regressed patient satisfaction on each LIWC language variable (in separate models) and allowed the intercepts to vary by resident physician, focusing first on language variables that have previously demonstrated a relationship with physician implicit bias. This yielded one significant linear relationship—patients whose physicians used more work-related language reported greater satisfaction, *b* = 0.31, *t*(285) = 1.98, *95% CI:* [0.00, 0.61]. This effect did not persist after corrections for multiple significance tests or when Hispanic identifying physicians were excluded from the analysis.

In an exploratory analysis, we estimated the same models for the remaining LIWC language variables (see Table C in S1 Table). Several additional relationships between resident language and patient satisfaction emerged; however, given the large number (75) of hypotheses tested, we controlled for the false discovery rate [52]. This approach is an alternative to family wise error corrections such as Bonferroni corrections and results in higher statistical power. After these corrections, only the effect of use of articles on patient satisfaction remained significant, *b* = 0.21, *t*(285) = 3.04, *95% CI*: [0.09, 0.34]. Patients whose physicians used more articles during their visits reported higher satisfaction. This effect remained significant whether or not Hispanic identifying physicians were included in the analysis. All relationships that reached statistical significance without correcting for Type 1 error inflation are described in S1 Appendix. Since these relationships may be the result of Type 1 errors they should be interpreted with caution. However, given the uniqueness of this dataset, we include these supplemental results for the reference of future researchers examining similar relationships.

Third, the quadratic relationship between implicit bias and patient satisfaction suggests that implicit bias relates to patient satisfaction primarily when physicians express higher levels of bias. Thus, we examined whether physician word choice might be related to patient satisfaction differently for physicians with differing levels of implicit bias. To test these relationships, we regressed patient satisfaction on physician bias, each language variable (separately), and their interaction. Once again, we began by examining language variables supported by previous literature. This initial approach resulted in two significant interaction effects: physician implicit bias interacted with language style matching, *b* = −5.71, *t*(283) = −2.34, *95% CI:* [−10.47, −0.94], and with physician use of 1^st^ person singular pronouns, *b* = −0.46, *t*(283) = −2.56, *95% CI:* [−0.81, −0.11]. These effects remained significant whether or not Hispanic identifying physicians were included in the analysis.

To examine these moderating effects, we calculated the simple effects of language at low (−1 SD from the mean), mean, and high (+1 SD from the mean) levels of physician implicit bias. The results of these simple effects tests can be found in Table 1. Both of these language variables demonstrated a negative and significant relationship with patient satisfaction only for physicians high in implicit bias. That is, for physicians high in implicit anti-Hispanic bias, greater language style matching and greater use of 1^st^ person singular pronouns was associated with lower levels of patient satisfaction.

**Table 1:** Simple effects for language X implicit bias interactions.

	Low Implicit Bias						Mean Implicit Bias					High Implicit Bias				
	b	df	t		95% CI		b	df	t	95% CI		b	df	t	95% CI	
1^st^ person singular pronouns	0.15	283	1.43		−0.06	0.36	−0.02	283	−0.24	−0.16	0.12	−0.19	283	−2.12	−0.36	−0.01
Auxiliary verbs	0.26	283	2.85		0.08	0.44	0.11	283	1.87	0.00	0.23	−0.04	283	−0.43	−0.20	0.12
Negations	0.48	283	2.65		0.13	0.83	0.18	283	1.56	−0.05	0.41	−0.11	283	−0.66	−0.44	0.22
Positive emotions	−0.08	283	−1.20		−0.22	0.05	0.01	283	0.15	−0.08	0.09	0.10	283	1.67	−0.02	0.21
Friend	−2.06	283	−2.63		−3.59	−0.53	−0.50	283	−0.89	−1.60	0.60	1.06	283	1.09	−0.83	2.95
Cognitive processes	0.09	283	1.67		−0.02	0.20	−0.02	283	−0.52	−0.09	0.05	−0.13	283	−2.52	−0.23	−0.03
Insight	0.34	283	2.05		0.02	0.66	0.09	283	0.76	−0.14	0.31	−0.16	283	−0.99	−0.48	0.16
Discrepancy	0.23	283	1.35		−0.10	0.56	−0.09	283	−0.77	−0.30	0.13	−0.40	283	−2.70	−0.69	−0.11
Differentiation	0.23	283	2.10		0.02	0.44	−0.01	283	−0.15	−0.16	0.14	−0.25	283	−2.37	−0.46	−0.04
Sexual	0.91	283	1.65		−0.17	1.99	0.12	283	0.32	−0.62	0.86	−0.67	283	−1.52	−1.53	0.19
Reward	−0.20	283	−1.41		−0.47	0.08	0.04	283	0.37	−0.15	0.23	0.27	283	2.00	0.01	0.53
Informal	−0.11	283	−2.74		−0.19	−0.03	−0.04	283	−1.66	−0.09	0.01	0.03	283	0.85	−0.03	0.09
Assent	−0.14	283	−2.51		−0.25	−0.03	−0.05	283	−1.42	−0.12	0.02	0.04	283	0.87	−0.05	0.13
Language Style Matching	1.53	283	1.18		−1.01	4.08	−0.59	283	−0.65	−2.36	1.18	−2.71	283	−2.15	−5.18	−0.25

*Note.* This table contains simple effects of language on patient satisfaction for physicians with at low (−1SD), mean, and high (+1SD) levels of implicit bias.

Again, we ran an exploratory analysis that examined parallel models for the remaining LIWC language variables. These effects are reported in Table D in S1 Table. Several relationships met the uncorrected standard for statistical significance and are described in S1 Appendix. After controlling for the false discovery rate, no interaction effect met the corrected criterion for statistical significance.

## Discussion

This study yields several novel findings regarding the interplay between physician implicit bias, word use, and patient satisfaction. First, the relationship between physician implicit bias and patient satisfaction was not linear as reported in previous research [21,53]; implicit bias was only significantly (and negatively) related to patient satisfaction when it was high compared to moderate or low. Whereas this is consistent with the assumption that physicians with high implicit bias communicate their negative attitudes and stereotypes through word choice, this quadratic effect could also indicate that Hispanic patients expect some level of prejudice and are, thus only impacted when physicians display high levels of prejudice [54]. Alternatively, this quadratic effect may suggest that physicians with lower and moderate levels of implicit bias are better able or more willing to control these biases. Indeed, in this study, physicians with higher levels of implicit bias tended to display lower levels of external motivation to control prejudice, suggesting that higher bias physicians may be less motivated to appear non-biased due to normative concerns.

Other possibilities for the difference in findings could be the target groups studied in the respective studies. Whereas Cooper and colleagues (2012) and Gonzalez and colleagues (2024) examined the relationship between anti-Black implicit bias and patient (or standardized patient) perceptions of care, the current research examined bias towards Hispanic patients. It is possible that bias towards Hispanic patients is different in nature or operates differently than specific anti-Black stereotypes about healthcare. That is, although previous research suggests that non-compliance stereotypes exist for both Black and Hispanic patients, it may be that other stereotypes are more relevant for predicting physician behaviors towards Hispanic patients; whereas non-compliance stereotypes are the most relevant for predicting behavior towards Black patients. Differences in the operationalization of implicit bias may have also led to the difference in findings. Cooper and colleagues (2012) also measured both implicit prejudice and implicit non-compliance stereotypes, but treated these measures as separate linear predictors of patient perceptions of care. Given the strong correlation between the implicit prejudice and stereotype measures in the current study, we aggregated these scores to form a single implicit bias measure when predicting patient satisfaction.

Finally, we note the ease with which people can correctly identify the race or ethnicity of a patient may play a role in how implicit bias impacts patient care. Research indicates that people are less accurate at correctly categorizing Hispanic compared to Black faces. In a clinical context, unless their ethnic identity is present on a chart or in the EHR, physicians, on average, may be less accurate at visually identifying Hispanic compared to Black patients. This may result in less consistent activation of prejudice and non-compliance stereotypes when physicians interact with Hispanic compared to Black patients. Further, more racially prejudiced individuals and those higher in race essentialism are more careful to correctly categorize others based on race/ethnicity [34,55,56]. Thus, it may also be that higher bias physicians are more likely to accurately identify the ethnicity of Hispanic patients and more susceptible to activation of negative associations than physicians with lower or moderate levels of anti-Hispanic implicit bias.

Our second set of findings demonstrated two relationships between physician language and patient satisfaction. Across levels of physician implicit bias, greater use of work related language and greater use of articles were associated with higher patient satisfaction, and these relationships did not depend on the physician’s level of implicit bias. Some previous research on work-related language has characterized use of work-related language as an indicator of focus on the task at hand [57,58] while other research shows that people who are made to feel powerful use more work-related language [31]. These findings present two potential explanations for the findings on work-related language and patient satisfaction. First, it may be that patients view physicians who use more work-related language as more task focused and respond favorably to this focused approach to patient care. Alternatively, patients view physicians who use more work-related language as being more powerful and, counterintuitively, prefer physicians who take a more hierarchical approach to patient care. Additional work should attempt to replicate this finding and examine these possible explanations.

There is very little research on the use of articles in spoken language so our explanation for this effect remains speculative. However, there is some evidence that when nouns are used to describe a person’s behavior rather than adjectives, they link that noun more strongly to the individual’s identity [59,60]. Since articles precede nouns, it may be that greater use of articles is indicative of physicians using more identity-focused language with their patients (e.g., “You’re not *an* exerciser” vs. “You haven’t been exercising much.”). It is unclear why such language use would be associated with positive patient impressions of care. Future work should examine the context in which articles are used during patient visits and explore additional explanations for this finding.

Our third set of findings found that two language variables interacted with physician implicit bias to predict patient satisfaction. For physicians high in implicit bias, greater use of first person singular pronouns and language style matching were associated with less patient satisfaction. Previous research has also found that the use of first-person singular pronouns relates to implicit bias and lower patient-centered care [61], possibly because the use of first-person pronouns signal that the physician is more focused on themselves than the patient. However, previous research on language style matching showed positive associations with positive interpersonal experiences [30,37]. Whereas our findings may seem at odds with previous literature, additional research suggests that impressions of language style matching may be complex. First, individuals with less power are more likely to match their language to high power individuals than the reverse and greater language style matching by low-power individuals is associated with more positive impressions [35]. Thus, the present findings might suggest that, when interacting with high bias physicians, Hispanic patients change their patterns of speech to match their physicians in an attempt to increase rapport, but resent the need to change their own behavior to seek better medical care. Alternatively, if physicians are changing their language to match that of their patients, patients may view this with distrust. In transactional relationships, individuals who engage in greater language style matching are viewed as more dishonest [42]. In the context of a medical visit, it may be that language style matching from a high-bias physician is seen as a cue for dishonesty or mistrust by Hispanic patients.

As a whole, these findings suggest that language may be one route by which physicians express implicit bias to their Hispanic patients, but this depends on the level of bias of their physician. Previous work suggests that implicit racial bias is associated with non-verbal and paraverbal behaviors [21,61], and the current findings suggest that this may primarily be the case for physicians at higher levels of implicit bias. However, the analysis of language by implicit bias interactions suggests that bias may be important for doctor-physician interactions in specific contexts. These moderated effects may arise for a few reasons. First, language use is contextual [62] and the language may be differentially predictive of interaction patterns in different situations. For example, verbal productivity scores in public, day-to-day contexts was predictive of extraversion, but the same verbal productivity scores generated from private speech were not, speaking to how communication contexts can modulate the psychological meaning of language markers [63]. Thus, the same words may be used in different contexts by high vs. low bias physicians and therefore may have differential implications for the overall evaluations of patients. Second, patient impressions are likely determined by multiple factors. For example, prosody impacts the interpretation of language, especially when language is positive [64]. Thus, seemingly innocuous word choice when viewed in the context of a transcript may communicate disdain or unfriendliness if communicated with a sarcastic tone. Alternatively, previous research suggests that non-verbal friendliness, which was also associated with implicit anti-Black bias, was positively associated with a Black interaction partner’s impression of a White participant [65]. Thus, it may be that the unique combination of both *what* a physician said and *how* they said it contributes to patients’ satisfaction with their outpatient visit.

Finally, the current study may have allowed for detection of these more nuanced effects due to its unique study design, in which several patient visits were nested under individual resident physicians. This approach, in contrast to approaches that measure only one patient visit per physician, may allow for more stable estimates of relationships between physician implicit bias and aspects of (statistically noisy) patient interactions. Future research should explore these possible mechanisms by examining the contexts in which physicians of different levels of bias are using different types of language and should explore whether verbal and non-verbal behaviors interact to influence patient outcomes.

### Practical implications

These findings, if supported by additional confirmatory tests, may have implications for ways to improve patient care. For example, physicians with less satisfied patients may carefully consider their word choice and communication patterns, particularly with regard to language style matching, first person singular pronouns, work-related language, and articles. We recommend carefully testing any training developed as a result of these (or any) findings before widespread implementation as some approaches, such as demonstrating the IAT, can have unintended negative consequences [66]. Notably, our study did not find that training directly related to patient satisfaction or moderated the implicit bias-patient satisfaction relationship. This could be because current training programs do not consider the specific behaviors that communicate bias to participants or could simply be a function of the diverse approaches to bias training on which participants based their responses to these items.

### Limitations and future directions

Although this work suggests linguistic pathways by which physicians communicate implicit bias to patients from stigmatized groups, these findings should be interpreted with caution. First, these findings are largely the result of exploratory analyses and correcting for the type 1 error inflation associated with a large number of statistical tests eliminates many of these significant effects. Although it is important to fully consider all possible information that can be gleaned from a field study involving highly specified research participants (i.e., physicians and their Hispanic patients) future studies should seek to confirm the tentative findings presented in this paper.

Second, it is important to note that whereas LIWC provides an efficient way to quantify language use, it does not capture the context in which such language is being used [67]. Although this research suggests, for example, that using more work-related language was associated with greater patient satisfaction for high-bias physicians, we do not yet have information on exactly what words or in what context such work-related language was being used. We have presented two possibilities for this relationship, but these explanations cannot be disentangled without further coding of these data. Future analyses may benefit from coding of physician language for greater specificity in how different categories of language are used in doctor-patient interactions. Additionally, the context of specific word use may be different in different languages. Although some patient visits in the current study were conducted with some amount of Spanish language the vast majority (73.5%) were conducted entirely in English, based on patient preference (Patients’ preferred language was recorded in the patient record system. Patients who preferred to speak Spanish either met with a physician who spoke Spanish, used translator provided by the clinic, or brought a friend/family member who could translate on their behalf.). This imbalance in language spoken prevented the analysis of differences in the effects of word choice in English vs. Spanish. Future work may benefit by examining whether (and in what ways) the context of the language used changes these relationships.

Finally, the present study is correlational, and although there is evidence that implicit bias and physician language are related to patient satisfaction, this does not necessarily mean that such a relationship is causal. It is possible that one or more third variables are responsible for the associations reported in this paper. For example, it may be that physicians who treat patients with greater courtesy [68] tend to be lower in implicit bias and use different types of language, but their courtesy is what truly influences patient perceptions of their medical care.

In sum, the present study adds to a growing body of evidence that patient group perceptions of medical care differ based on the individual biases and behaviors of their doctors. Importantly, we extend this evidence to a new group: Hispanic patients. This paper provides several avenues for future work, namely confirming relationships found through exploratory analysis in the present work and exploring the context in which certain types of language are used in the context of medical care.

## Supporting information

S1 Table Contains Supplemental Tables A-D. (DOCX)

S1 Appendix Contains a description of additional analyses without accounting for Type 1 error inflation. (DOCX)

S1 File Inclusivity in global research questionnaire. (DOCX)

## References

[1] Fiscella K, Franks P, Doescher MP, Saver BG. Disparities in health care by race, ethnicity, and language among the insured: findings from a national sample. Med Care. 2002;40(1):52–9. doi: 10.1097/00005650-200201000-00007 11748426

[2] Life expectancy by county, race, and ethnicity in the USA, 2000–19: a systematic analysis of health disparities. The Lancet. 2022;400(10345):25–38. 10.1016/S0140-6736(22)00876-5 PMC9256789 35717994

[3] Ramprasad A, Qureshi F, Lee BR, Jones BL. The relationship between structural racism and COVID-19 related health disparities across 10 metropolitan cities in the United States. J Natl Med Assoc. 2022;114(3):265–73. doi: 10.1016/j.jnma.2022.01.008 35221074 PMC8872840

[4] FitzGerald C, Hurst S. Implicit bias in healthcare professionals: a systematic review. BMC Med Ethics. 2017;18(1):19. doi: 10.1186/s12910-017-0179-8 28249596 PMC5333436

[5] Nosek BA, Smyth FL, Hansen JJ, Devos T, Lindner NM, Ranganath KA, et al. Pervasiveness and correlates of implicit attitudes and stereotypes. Eur Rev Soc Psychol. 2007;18(1):36–88. doi: 10.1080/10463280701489053

[6] Greenwald AG, Banaji MR. Implicit social cognition: attitudes, self-esteem, and stereotypes. Psychol Rev. 1995;102(1):4–27. doi: 10.1037/0033-295x.102.1.4 7878162

[7] Liang J, Wolsiefer K, Zestcott CA, Chase D, Stone J. Implicit bias toward cervical cancer: provider and training differences. Gynecol Oncol. 2019;153(1):80–6. doi: 10.1016/j.ygyno.2019.01.013 30739720

[8] Maina IW, Belton TD, Ginzberg S, Singh A, Johnson TJ. A decade of studying implicit racial/ethnic bias in healthcare providers using the implicit association test. Soc Sci Med. 2018;199:219–29. doi: 10.1016/j.socscimed.2017.05.009 28532892

[9] Sriram N, Mills J, Lang E, Dickson HK, Hamann HA, Nosek BA, et al. Attitudes and stereotypes in lung cancer versus breast cancer. PLoS One. 2015;10(12):e0145715. doi: 10.1371/journal.pone.0145715 26698307 PMC4689531

[10] Stringer KL, Turan B, McCormick L, Durojaiye M, Nyblade L, Kempf M-C, et al. HIV-related stigma among healthcare providers in the deep south. AIDS Behav. 2016;20(1):115–25. doi: 10.1007/s10461-015-1256-y 26650383 PMC4718797

[11] VanPuymbrouck L, Friedman C, Feldner H. Explicit and implicit disability attitudes of healthcare providers. Rehabil Psychol. 2020;65(2):101–12. doi: 10.1037/rep0000317 32105109 PMC9534792

[12] Zestcott CA, Spece L, McDermott D, Stone J. Health care providers’ negative implicit attitudes and stereotypes of American Indians. J Racial Ethn Health Disparities. 2021;8(1):230–6. doi: 10.1007/s40615-020-00776-w 32445056

[13] Blair IV, Steiner JF, Fairclough DL, Hanratty R, Price DW, Hirsh HK, et al. Clinicians’ implicit ethnic/racial bias and perceptions of care among Black and Latino patients. Ann Fam Med. 2013;11(1):43–52. doi: 10.1370/afm.1442 23319505 PMC3596038

[14] Haider AH, Sexton J, Sriram N, Cooper LA, Efron DT, Swoboda S, et al. Association of unconscious race and social class bias with vignette-based clinical assessments by medical students. J Am Med Assoc. 2011;306(9):942–51. doi: 10.1001/jama.2011.1248 21900134 PMC3684149

[15] Haider AH, Schneider EB, Sriram N, Dossick DS, Scott VK, Swoboda SM, et al. Unconscious race and class bias: its association with decision making by trauma and acute care surgeons. J Trauma Acute Care Surg. 2014;77(3):409–16. doi: 10.1097/TA.0000000000000392 25159243

[16] Hirsh AT, Hollingshead NA, Ashburn-Nardo L, Kroenke K. The interaction of patient race, provider bias, and clinical ambiguity on pain management decisions. J Pain. 2015;16(6):558–68. doi: 10.1016/j.jpain.2015.02.003 25828370 PMC4456233

[17] Oliver MN, Wells KM, Joy-Gaba JA, Hawkins CB, Nosek BA. Do physicians’ implicit views of African Americans affect clinical decision making? J Am Board Fam Med. 2014;27(2):177–88. doi: 10.3122/jabfm.2014.02.120314 24610180

[18] Puumala SE, Burgess KM, Kharbanda AB, Zook HG, Castille DM, Pickner WJ, et al. The role of bias by emergency department providers in care for American Indian children. Med Care. 2016;54(6):562–9. doi: 10.1097/MLR.0000000000000533 26974675 PMC4865441

[19] Sabin JA, Rivara FP, Greenwald AG. Physician implicit attitudes and stereotypes about race and quality of medical care. Med Care. 2008;46(7):678–85. doi: 10.1097/MLR.0b013e3181653d58 18580386

[20] Shapiro N, Wachtel EV, Bailey SM, Espiritu MM. Implicit physician biases in periviability counseling. J Pediatr. 2018;197:109–115.e1. doi: 10.1016/j.jpeds.2018.01.070 29571927

[21] Cooper LA, Roter DL, Carson KA, Beach MC, Sabin JA, Greenwald AG, et al. The Associations of Clinicians’ implicit attitudes about race with medical visit communication and patient ratings of interpersonal care. Am J Public Health. 2012;102(5):979–87. doi: 10.2105/ajph.2011.300558 22420787 PMC3483913

[22] Penner LA, Dovidio JF, Gonzalez R, Albrecht TL, Chapman R, Foster T, et al. The effects of oncologist implicit racial bias in racially discordant oncology interactions. J Clin Oncol. 2016;34(24):2874–80. doi: 10.1200/jco.2015.66.3658 27325865 PMC5012663

[23] Hausmann LRM, Myaskovsky L, Niyonkuru C, Oyster ML, Switzer GE, Burkitt KH, et al. Examining implicit bias of physicians who care for individuals with spinal cord injury: a pilot study and future directions. J Spinal Cord Med. 2015;38(1):102–10. doi: 10.1179/2045772313Y.0000000184 24621034 PMC4293524

[24] Lopez CF. Hispanic Americans’ Trust in and Engagement With Science. Pew Research Center. 2022. [cited 2025 Oct 7]. Available from: https://www.pewresearch.org/science/2022/06/14/hispanic-americans-trust-in-and-engagement-with-science/

[25] Dovidio JF, Gaertner SL, Pearson AR. Aversive Racism and Contemporary Bias. The Cambridge Handbook of the Psychology of Prejudice. New York, NY, US: Cambridge University Press; 2016. pp. 267–94. doi: 10.1017/9781316161579.012

[26] Pearson AR, Dovidio JF, Gaertner SL. The nature of contemporary prejudice: insights from aversive racism. Soc Pers Psychol Compass. 2009;3(3):314–38. doi: 10.1111/j.1751-9004.2009.00183.x

[27] Penner LA, Dovidio JF, West TV, Gaertner SL, Albrecht TL, Dailey RK, et al. Aversive racism and medical interactions with Black patients: a field study. J Exp Soc Psychol. 2010;46(2):436–40. doi: 10.1016/j.jesp.2009.11.004 20228874 PMC2835170

[28] Ashokkumar A, Pennebaker JW. Tracking group identity through natural language within groups. PNAS Nexus. 2022;1(2):1–9. doi: 10.1093/pnasnexus/pgad457 PMC9229362 35774418

[29] Chung CK, Pennebaker JW. Revealing dimensions of thinking in open-ended self-descriptions: An automated meaning extraction method for natural language. J Res Personal. 2008;42(1):96–132. doi: 10.1016/j.jrp.2007.04.006 PMC2542899 18802499

[30] Ireland ME, Slatcher RB, Eastwick PW, Scissors LE, Finkel EJ, Pennebaker JW. Language style matching predicts relationship initiation and stability. Psychol Sci. 2010;22(1):39–44. doi: 10.1177/0956797610392928 21149854

[31] Livingston TN, Vik TA, Singer J. Relationships between power, communication about work and sex, and emotion expression: a linguistic inquiry and word count analysis. Psychol Rep. 2024;127(3):1408–28. doi: 10.1177/00332941221137243 36302733

[32] Carnaghi A, Maass A, Gresta S, Bianchi M, Cadinu M, Arcuri L. Nomina sunt omina: on the inductive potential of nouns and adjectives in person perception. J Pers Soc Psychol. 2008;94(5):839–59. doi: 10.1037/0022-3514.94.5.839 18444742

[33] Carey AL, Brucks MS, Küfner ACP, Holtzman NS, Große Deters F, Back MD, et al. Narcissism and the use of personal pronouns revisited. J Pers Soc Psychol. 2015;109(3):e1–15. doi: 10.1037/pspp0000029 25822035

[34] Mandalaywala TM, Amodio DM, Rhodes M. Essentialism promotes racial prejudice by increasing endorsement of social hierarchies. Soc Psychol Personal Sci. 2018;9(4):461–9. doi: 10.1177/1948550617707020 33163145 PMC7643920

[35] Muir K, Joinson A, Cotterill R, Dewdney N. Linguistic style accommodation shapes impression formation and rapport in computer-mediated communication. J Lang Soc Psychol. 2017;36(5):525–48. doi: 10.1177/0261927x17701327

[36] Muir K, Joinson A, Cotterill R, Dewdney N. Characterizing the Linguistic Chameleon: personal and social correlates of linguistic style accommodation. Hum Commun Res. 2016;42(3):462–84. doi: 10.1111/hcre.12083

[37] Romero DM, Swaab RI, Uzzi B, Galinsky AD. Mimicry is presidential: linguistic style matching in presidential debates and improved polling numbers. Pers Soc Psychol Bull. 2015;41(10):1311–9. doi: 10.1177/0146167215591168 26195626

[38] Williams-Baucom KJ, Atkins DC, Sevier M, Eldridge Ka, Christensen A. “You” and “I” need to talk about “us”: Linguistic patterns in marital interactions. Pers Relationsh. 2010;17(1):41–56. doi: 10.1111/j.1475-6811.2010.01251.x

[39] Hagiwara N, Slatcher RB, Eggly S, Penner LA. Physician racial bias and word use during racially discordant medical interactions. Health Commun. 2017;32(4):401–8. doi: 10.1080/10410236.2016.1138389 27309596 PMC5161737

[40] Wolsiefer KJ, Mehl M, Moskowitz GB, Cagno CK, Zestcott CA, Tejeda-Padron A, et al. Investigating the relationship between resident physician implicit bias and language use during a clinical encounter with Hispanic patients. Health Commun. 2023;38(1):124–32. doi: 10.1080/10410236.2021.19367526 34130567 PMC9524003

[41] Hagiwara N, Penner LA, Gonzalez R, Eggly S, Dovidio JF, Gaertner SL, et al. Racial attitudes, physician-patient talk time ratio, and adherence in racially discordant medical interactions. Soc Sci Med. 2013;87:123–31. doi: 10.1016/j.socscimed.2013.03.016 23631787 PMC3677202

[42] Ludwig S, van Laer T, de Ruyter K, Friedman M. Untangling a web of lies: exploring automated detection of deception in computer-mediated communication. J Manag Inf Syst. 2016;33(2):511–41. doi: 10.1080/07421222.2016.1205927

[43] Pennebaker JW, Boyd RL, Jordan K, Blackburn K. The development and psychometric properties of LIWC2015. 2015.

[44] Spybrook J, Raudenbush SW, Liu XF, Congdon R, Martinez A. Optimal design for longitudinal and multilevel research: Documentation for the “Optimal Design” software. Surv Res Cent Inst Soc Res Univ Mich. 2006.

[45] Greenwald AG, Nosek BA, Banaji MR. Understanding and using the implicit association test: I. An improved scoring algorithm. J Pers Soc Psychol. 2003;85(2):197–216. doi: 10.1037/0022-3514.85.2.197 12916565

[46] Plant EA, Devine PG. Internal and external motivation to respond without prejudice. J Pers Soc Psychol. 1998;75(3):811–32. doi: 10.1037/0022-3514.75.3.811 16055643

[47] Davis MH. Measuring individual differences in empathy: evidence for a multidimensional approach. J Pers Soc Psychol. 1983;44(1):113–26. doi: 10.1037/0022-3514.44.1.113

[48] Neel R, Shapiro JR. Is racial bias malleable? Whites’ lay theories of racial bias predict divergent strategies for interracial interactions. J Pers Soc Psychol. 2012;103(1):101–20. doi: 10.1037/a0028237 22564011

[49] Dehghani M, Boyd RL. Handbook of Language Analysis in Psychology. New York, NY, US: Guilford Publications; 2022. pp. 650.

[50] Ireland ME, Pennebaker JW. Language style matching in writing: synchrony in essays, correspondence, and poetry. J Pers Soc Psychol. 2010;99(3):549–71. doi: 10.1037/a0020386 20804263

[51] Bierstetel SJ, Farrell AK, Briskin JL, Harvey MW, Gable SL, Ha T, et al. Associations between language style matching and relationship commitment and satisfaction: an integrative data analysis. J Soc Pers Relationsh. 2020;37(8–9):2459–81. doi: 10.1177/0265407520923754

[52] Benjamini Y, Hochberg Y. Controlling the false discovery rate: a practical and powerful approach to multiple testing. J R Stat Soc Ser B Methodol. 1995;57(1):289–300.

[53] Gonzalez CM, Ark TK, Fisher MR, Marantz PR, Burgess DJ, Milan F, et al. Racial implicit bias and communication among physicians in a simulated environment. JAMA Netw Open. 2024;7(3):e242181. doi: 10.1001/jamanetworkopen.2024.2181 38506811 PMC10955368

[54] Greer TM, Brondolo E, Brown P. Systemic racism moderates effects of provider racial biases on adherence to hypertension treatment for African Americans. Health Psychol. 2014;33(1):35–42. doi: 10.1037/a0032777 23730720

[55] Blascovich J, Wyer NA, Swart LA, Kibler JL. Racism and racial categorization. J Pers Soc Psychol. 1997;72(6):1364–72. doi: 10.1037/0022-3514.72.6.1364

[56] Chao MM, Hong Y, Chiu C. Essentializing race: its implications on racial categorization. J Pers Soc Psychol. 2013;104(4):619–34. doi: 10.1037/a0031332 23397967

[57] Anderson AA. Expressions of resilience: social media responses to a flooding event. Risk Anal. 2021;41(9):1600–13. doi: 10.1111/risa.13639 33190308

[58] Licorish SA, MacDonell SG. Understanding the attitudes, knowledge sharing behaviors and task performance of core developers: a longitudinal study. Inf Softw Technol. 2014;56(12):1578–96. doi: 10.1016/j.infsof.2014.02.004

[59] Bryan CJ, Master A, Walton GM. “Helping” versus “being a helper”: invoking the self to increase helping in young children. Child Dev. 2014;85(5):1836–42. doi: 10.1111/cdev.12244 24779480

[60] Bryan CJ, Adams GS, Monin B. When cheating would make you a cheater: implicating the self prevents unethical behavior. J Exp Psychol Gen. 2013;142(4):1001–5. doi: 10.1037/a0030655 23127418

[61] Hagiwara N, Dovidio JF, Eggly S, Penner LA. The effects of racial attitudes on affect and engagement in racially discordant medical interactions between non-Black physicians and Black patients. Group Process Intergroup Relat. 2016;19(4):509–27. doi: 10.1177/1368430216641306 27642254 PMC5019493

[62] Collins KA, Clément R. Language and prejudice: direct and moderated dffects. J Language Soc Psychol. 2012;31(4):376–96. doi: 10.1177/0261927x12446611

[63] Wernick N. How taking a word for a word can be problematic: context-dependent linguistic markers of extraversion and neuroticism. J Methods Meas Soc Sci. 2013;3(2). doi: 10.2458/v3i2.16477

[64] Mauchand M, Vergis N, Pell MD. Irony, prosody, and social impressions of affective stance. Discourse Processes. 2019;57(2):141–57. doi: 10.1080/0163853x.2019.1581588

[65] Dovidio JF, Kawakami K, Gaertner SL. Implicit and explicit prejudice and interracial interaction. J Pers Soc Psychol. 2002;82(1):62–8. doi: 10.1037//0022-3514.82.1.62 11811635

[66] Lofaro N, Irving LH, Ratliff KA. Defensiveness toward IAT feedback predicts willingness to engage in anti-bias behaviors. Pers Soc Psychol Bull. 2025;51(8):1411–30. doi: 10.1177/01461672231219948 38179987

[67] Eichstaedt JC, Kern ML, Yaden DB, Schwartz HA, Giorgi S, Park G, et al. Closed- and open-vocabulary approaches to text analysis: a review, quantitative comparison, and recommendations. Psychol Methods. 2021;26(4):398–427. doi: 10.1037/met0000349 34726465

[68] Comstock LM, Hooper EM, Goodwin JM, Goodwin JS. Physician behaviors that correlate with patient satisfaction. J Med Educ. 1982;57(2):105–12. doi: 10.1097/00001888-198202000-00005 7057429

