# Supplementary material for: Examining the relationships among physician implicit bias, language, and Hispanic patient satisfaction
Source: PLoS One. 2025 Dec 31;20(12):e0338699. doi: 10.1371/journal.pone.0338699 (PMC12755806; doi:10.1371/journal.pone.0338699)
Supplement: S1 Table — (DOCX) [file pone.0338699.s001.docx]

| **Table A** | | | |  |
| --- | --- | --- | --- | --- |
| **LIWC Language Category Examples & Descriptions** | | | |  |
| Category | Examples/Description | Category | Examples/Description | |
| Function Words | it, to, no, very | Hear | listen, hearing | |
| Personal pronouns | I, them, itself | Feel | feels, touch | |
| 1st person singular | I, me, mine | Biological processes | eat, blood, pain | |
| 1st person plural | we, us, our | Body | cheek, hands, spit | |
| 2nd person | you, your, thou | Health | clinic, flu, pill | |
| 3rd person singular | she, her, him | Sexual | horny, love, incest | |
| 3rd person plural | they, their, they'd | Ingestion | dish, eat, pizza | |
| Impersonal pronouns | it, it's, those | Affiliation | ally, friend, social | |
| Articles | a, an, the | Achievement | win, success, better | |
| Prepositions | to, with, above | Power | superior, bully | |
| Auxiliary verbs | am, will, have | Reward | take, prize, benefit | |
| Common adverbs | very, really | Risk | danger, doubt | |
| Conjunctions | and, but, whereas | Drives | Words from the categories: affiliation, achievement, power, reward, risk | |
| Negations | no, not, never | Past focus | ago, did, talked | |
| Common verbs | eat, come, carry | Present focus | today, is, now | |
| Common adjectives | free, happy, long | Future focus | may, will, soon | |
| Comparisons | greater, best, after | Time orientations | Words from the categories: past focus, present focus, future focus | |
| Interrogatives | how, when, what | Relativity | area, bend, exit | |
| Numbers | second, thousand | Motion | arrive, car, go | |
| Quantifiers | few, many, much | Space | down, in, thin | |
| Affective processes | happy, cried | Time | end, until, season | |
| Positive emotion | love, nice, sweet | Work | job, majors, xerox | |
| Negative emotion | hurt, ugly, nasty | Leisure | cook, chat, movie | |
| Anxiety | worried, fearful | Home | kitchen, landlord | |
| Anger | hate, kill, annoyed | Money | audit, cash, owe | |
| Sadness | crying, grief, sad | Religion | altar, church | |
| Social processes | mate, talk, they | Death | bury, coffin, kill | |
| *Note.* The list of categories, example words, and descriptions were taken from the LIWC 2015 manual (LIWC, 2015). More detailed information about these categories, including internal consistency statistics, can be found in the manual. | | | | |
| **Table A (continued)** | | | | |
| **LIWC Language Category Examples & Descriptions** | | | | |
| Category | Examples/Description | Category | Examples/Description | |
| Male references | boy, his, dad | Nonfluencies | er, hm, umm | |
| Cognitive processes | cause, know, ought | Fillers | "I mean", "you know" | |
| Family | daughter, dad, aunt | Swear words | fuck, damn, shit | |
| Friends | buddy, neighbor | Netspeak | btw, lol, thx | |
| Female references | girl, her, mom | Assent | agree, OK, yes | |
| Insight | think, know | Informal language | Words from the categories: swear words, netspeak, assent, nonfluences, and fillers | |
| Causation | because, effect | Analytical thinking | Standardized score calculated from existing LIWC categories. Higher values indicate patterns of language related to more formal, logical, and hierarchical thinking patterns. | |
| Discrepancy | should, would | Clout | Standardized score calculated from existing LIWC categories. Higher values indicate patterns of language related to higher social status, confidence, or leadership. | |
| *Note.* The list of categories, example words, and descriptions were taken from the LIWC 2015 manual (LIWC, 2015). More detailed information about these categories, including internal consistency statistics, can be found in the manual. | | | | |
| **Table A (continued)** | | | | |
| **LIWC Language Category Examples & Descriptions** | | | | |
| Category | Examples/Description | Category | Examples/Description | |
| Tentative | maybe, perhaps | Authentic | Standardized score calculated from existing LIWC categories. Higher values indicate patterns of language related to higher humility, vulnerability and honesty. | |
| Certainty | always, never | Emotional tone | A combination of the positive and negative emotion variables. A higher score indicates a more positive emotional tone. Scores above 50 indicate a more positive tone than negative. Scores below 50 indicate a more negative tone than positive. | |
| Differentiation | hasn't, but, else | Words/sentence | Number of words per sentence | |
| Perceptual processes | look, heard, feeling | Words > 6 letters | Number of words with greater than 6 letters. | |
| See | view, saw, seen | Word count | Total words | |
| *Note.* The list of categories, example words, and descriptions were taken from the LIWC 2015 manual (LIWC, 2015). More detailed information about these categories, including internal consistency statistics, can be found in the manual. | | | | |

| **Table B.** Quadratic Effects of Implicit Bias on Resident Language Use | | | | | |
| --- | --- | --- | --- | --- | --- |
| Language Variable | b | df | t | 95% CI | |
| Function words | 0.67 | 44.59 | 0.57 | -1.62 | 2.95 |
| Pronouns | 1.14 | 46.02 | 1.36 | -0.49 | 2.77 |
| Personal pronouns | 1.14 | 47.38 | 1.52 | -0.31 | 2.59 |
| 1^st^ person singular pronouns* | 0.30 | 48.37 | 0.64 | -0.60 | 1.19 |
| 1^st^ person plural pronouns | 0.18 | 48.76 | 0.48 | -0.54 | 0.90 |
| 2^nd^ person pronouns | 0.31 | 46.91 | 0.42 | -1.11 | 1.73 |
| 3^rd^ person singular pronouns | 0.13 | 41.06 | 0.64 | -0.25 | 0.51 |
| 3^rd^ person plural pronouns | 0.24 | 44.49 | 1.56 | -0.06 | 0.54 |
| Impersonal pronouns | 0.00 | 47.69 | 0.00 | -1.22 | 1.21 |
| Articles | -0.45 | 47.64 | -1.00 | -1.33 | 0.42 |
| Prepositions* | -0.44 | 47.29 | -0.75 | -1.60 | 0.71 |
| Auxiliary verbs | 0.52 | 44.78 | 0.96 | -0.54 | 1.58 |
| Common adverbs | -0.15 | 48.01 | -0.33 | -1.04 | 0.74 |
| Conjunctions | 0.12 | 49.17 | 0.17 | -1.26 | 1.50 |
| Negations | -0.19 | 44.77 | -0.79 | -0.65 | 0.27 |
| Common verbs | 0.24 | 46.45 | 0.30 | -1.30 | 1.77 |
| Common adjectives | 0.44 | 46.55 | 1.11 | -0.32 | 1.20 |
| Comparisons | 0.01 | 43.30 | 0.04 | -0.47 | 0.49 |
| Interrogatives* | -0.03 | 46.12 | -0.14 | -0.44 | 0.38 |
| Numbers | -0.37 | 38.10 | -0.92 | -1.14 | 0.40 |
| Quantifiers | 0.14 | 45.59 | 0.51 | -0.40 | 0.68 |
| Affective processes | 0.70 | 48.10 | 0.88 | -0.84 | 2.24 |
| Positive emotions | 1.01 | 46.97 | 1.29 | -0.51 | 2.54 |
| Negative emotions | -0.27 | 47.71 | -1.35 | -0.67 | 0.12 |
| Anxiety words* | -0.10 | 42.93 | -1.12 | -0.28 | 0.07 |
| Anger words | 0.01 | 47.65 | 0.57 | -0.03 | 0.06 |
| Sadness words | -0.11 | 46.08 | -1.43 | -0.27 | 0.04 |
| Social Processes | 0.67 | 47.45 | 0.85 | -0.85 | 2.18 |
| Family words | 0.03 | 288.00 | 0.57 | -0.08 | 0.15 |
| Friend | 0.01 | 51.36 | 0.21 | -0.06 | 0.08 |
| Female references | 0.09 | 40.68 | 0.49 | -0.26 | 0.44 |
| Male references | 0.00 | 39.15 | -0.04 | -0.24 | 0.23 |
| Cognitive processes | 0.69 | 46.94 | 0.82 | -0.94 | 2.32 |
| Insight words | 0.09 | 42.27 | 0.37 | -0.37 | 0.54 |
| Causation words | -0.26 | 44.96 | -1.44 | -0.60 | 0.09 |
| Discrepancy words | 0.27 | 46.13 | 0.96 | -0.27 | 0.81 |
| Tentative words | 0.26 | 47.22 | 0.46 | -0.82 | 1.34 |
| *Note.* Each row represents the quadratic relationship between resident bias and the specified LIWC language variable. * indicates a variable examined in the initial analysis. | | | | | |
| **Table S2, continued** | | | | | |
| Language Variable | b | df | t | 95% CI | |
| Certainty words | 0.37 | 44.49 | 1.50 | -0.11 | 0.85 |
| Differentiation | 0.08 | 45.22 | 0.19 | -0.72 | 0.87 |
| Perceptual processes | 0.13 | 48.31 | 0.43 | -0.45 | 0.70 |
| See | 0.26 | 46.32 | 1.27 | -0.14 | 0.66 |
| Hear | 0.03 | 46.24 | 0.36 | -0.15 | 0.22 |
| Feel | -0.07 | 48.00 | -0.29 | -0.56 | 0.41 |
| Biological | -0.41 | 46.32 | -0.69 | -1.55 | 0.74 |
| Body | -0.24 | 48.33 | -0.88 | -0.79 | 0.30 |
| Health | 0.04 | 45.80 | 0.11 | -0.70 | 0.78 |
| Sexual | 0.11 | 40.42 | 1.75 | -0.01 | 0.23 |
| Ingestion | -0.17 | 41.84 | -0.69 | -0.64 | 0.30 |
| Drives | 0.03 | 48.86 | 0.05 | -1.10 | 1.16 |
| Affiliation | 0.19 | 48.63 | 0.47 | -0.60 | 0.99 |
| Achievement | 0.27 | 46.87 | 1.85 | -0.01 | 0.54 |
| Power | -0.22 | 51.10 | -1.40 | -0.52 | 0.08 |
| Reward | 0.11 | 47.71 | 0.36 | -0.49 | 0.72 |
| Risk | -0.07 | 45.68 | -0.69 | -0.27 | 0.13 |
| Past-focused | -0.14 | 45.85 | -0.35 | -0.92 | 0.64 |
| Present-focused | 0.46 | 45.68 | 0.53 | -1.20 | 2.12 |
| Future-focused* | 0.17 | 44.56 | 0.65 | -0.34 | 0.69 |
| Relativity (motion, space, time)* | -0.80 | 45.20 | -1.10 | -2.20 | 0.61 |
| Motion | -0.13 | 44.48 | -0.60 | -0.57 | 0.30 |
| Space | -0.30 | 47.55 | -0.58 | -1.31 | 0.71 |
| Time* | -0.37 | 42.92 | -0.82 | -1.25 | 0.51 |
| Work* | 0.20 | 48.90 | 1.19 | -0.12 | 0.51 |
| Leisure | 0.08 | 40.38 | 0.90 | -0.09 | 0.24 |
| Home | -0.04 | 41.08 | -0.69 | -0.17 | 0.08 |
| Money | -0.04 | 46.17 | -0.77 | -0.14 | 0.06 |
| Religion | -0.01 | 47.77 | -0.56 | -0.04 | 0.02 |
| Death | -0.01 | 48.87 | -0.59 | -0.04 | 0.02 |
| Informal | 0.91 | 48.16 | 0.59 | -2.08 | 3.91 |
| Swear | -0.01 | 38.34 | -1.07 | -0.02 | 0.01 |
| Assent | 0.75 | 48.24 | 0.78 | -1.13 | 2.64 |
| Non-fluences | 0.04 | 45.68 | 0.06 | -1.25 | 1.32 |
| Filler words | 0.12 | 50.23 | 0.34 | -0.57 | 0.82 |
| Language Style Matching* | 0.02 | 50.42 | 0.60 | -0.04 | 0.08 |
| *Note.* Each row represents the quadratic relationship between resident bias and the specified LIWC language variable. * indicates a variable examined in the initial analysis. | | | | | |

| **Table C.** Resident Language and Patient Satisfaction | | | | | |
| --- | --- | --- | --- | --- | --- |
| Language Variable | b | df | t | 95% CI | |
| Function words | 0.02 | 285 | 0.76 | -0.03 | 0.08 |
| Pronouns | -0.01 | 285 | -0.35 | -0.10 | 0.07 |
| Personal pronouns | -0.09 | 285 | -1.94 | -0.17 | 0.00 |
| 1^st^ person singular pronouns* | -0.07 | 285 | -0.92 | -0.20 | 0.07 |
| 1^st^ person plural pronouns | 0.20 | 285 | 2.14 | 0.02 | 0.39 |
| 2^nd^ person pronouns | -0.09 | 285 | -1.93 | -0.18 | 0.00 |
| 3^rd^ person singular pronouns | -0.11 | 285 | -0.80 | -0.36 | 0.15 |
| 3^rd^ person plural pronouns | -0.18 | 285 | -1.04 | -0.51 | 0.16 |
| Impersonal pronouns | 0.09 | 285 | 1.79 | -0.01 | 0.18 |
| Articles | 0.21 | 285 | 3.04 | 0.07 | 0.34 |
| Prepositions* | -0.03 | 285 | -0.62 | -0.14 | 0.07 |
| Auxiliary verbs | 0.10 | 285 | 1.59 | -0.02 | 0.21 |
| Common adverbs | -0.05 | 285 | -0.75 | -0.17 | 0.08 |
| Conjunctions | -0.01 | 285 | -0.19 | -0.11 | 0.09 |
| Negations | 0.16 | 285 | 1.33 | -0.07 | 0.39 |
| Common verbs | 0.01 | 285 | 0.18 | -0.08 | 0.09 |
| Common adjectives | 0.10 | 285 | 1.10 | -0.07 | 0.27 |
| Comparisons | 0.10 | 285 | 0.94 | -0.11 | 0.32 |
| Interrogatives | -0.08 | 285 | -0.58 | -0.37 | 0.20 |
| Numbers | 0.04 | 285 | 0.68 | -0.08 | 0.16 |
| Quantifiers | 0.01 | 285 | 0.06 | -0.20 | 0.21 |
| Affective processes | 0.02 | 285 | 0.47 | -0.06 | 0.10 |
| Positive emotions | 0.02 | 285 | 0.40 | -0.07 | 0.10 |
| Negative emotions | 0.04 | 285 | 0.36 | -0.17 | 0.25 |
| Anxiety words* | 0.01 | 285 | 0.04 | -0.46 | 0.48 |
| Anger words | -0.93 | 285 | -1.07 | -2.62 | 0.77 |
| Sadness words | 0.06 | 285 | 0.20 | -0.51 | 0.62 |
| Social Processes | -0.05 | 285 | -1.17 | -0.13 | 0.03 |
| Family words | 0.01 | 285 | 0.03 | -0.66 | 0.68 |
| Friend | -0.76 | 285 | -1.37 | -1.85 | 0.33 |
| Female references | 0.04 | 285 | 0.27 | -0.26 | 0.34 |
| Male references | -0.31 | 285 | -1.54 | -0.70 | 0.08 |
| Cognitive processes | -0.03 | 285 | -0.77 | -0.10 | 0.04 |
| Insight words | 0.08 | 285 | 0.71 | -0.15 | 0.31 |
| Causation words | 0.15 | 285 | 1.08 | -0.12 | 0.42 |
| Discrepancy words | -0.13 | 285 | -1.15 | -0.35 | 0.09 |
| Tentative words | -0.11 | 285 | -1.80 | -0.22 | 0.01 |
| *Note.* Each row represents the quadratic relationship between resident bias and the specified LIWC language variable. * indicates a variable examined in the initial analysis. | | | | | |
| **Table C, continued** | | | | | |
| Language Variable | b | df | t | 95% CI | |
| Certainty words | -0.07 | 285.00 | -0.59 | -0.30 | 0.16 |
| Differentiation | -0.02 | 285.00 | -0.32 | -0.18 | 0.13 |
| Perceptual processes | -0.16 | 285.00 | -2.04 | -0.31 | -0.01 |
| See | -0.23 | 285.00 | -1.88 | -0.47 | 0.01 |
| Hear | -0.27 | 285.00 | -1.06 | -0.76 | 0.23 |
| Feel | -0.08 | 285.00 | -0.78 | -0.28 | 0.12 |
| Biological | -0.02 | 285.00 | -0.43 | -0.12 | 0.08 |
| Body | -0.08 | 285.00 | -0.84 | -0.27 | 0.11 |
| Health | -0.05 | 285.00 | -0.69 | -0.19 | 0.09 |
| Sexual | -0.10 | 285.00 | -0.26 | -0.82 | 0.63 |
| Ingestion | 0.10 | 285.00 | 0.89 | -0.11 | 0.30 |
| Drives | 0.14 | 285.00 | 2.60 | 0.04 | 0.25 |
| Affiliation | 0.19 | 285.00 | 2.16 | 0.02 | 0.35 |
| Achievement | 0.07 | 285.00 | 0.40 | -0.27 | 0.40 |
| Power | 0.03 | 285.00 | 0.18 | -0.26 | 0.32 |
| Reward | 0.06 | 285.00 | 0.57 | -0.14 | 0.25 |
| Risk | 0.49 | 285.00 | 1.77 | -0.05 | 1.03 |
| Past-focused | -0.06 | 285.00 | -0.80 | -0.22 | 0.09 |
| Present-focused | 0.06 | 285.00 | 1.30 | -0.03 | 0.14 |
| Future-focused* | -0.12 | 285.00 | -1.24 | -0.31 | 0.07 |
| Relativity (motion, space, time)* | -0.01 | 285.00 | -0.18 | -0.08 | 0.07 |
| Motion | -0.03 | 285.00 | -0.29 | -0.25 | 0.19 |
| Space | 0.02 | 285.00 | 0.26 | -0.10 | 0.13 |
| Time* | -0.04 | 285.00 | -0.67 | -0.15 | 0.07 |
| Work* | 0.31 | 285.00 | 1.98 | 0.00 | 0.61 |
| Leisure | 0.12 | 285.00 | 0.48 | -0.38 | 0.62 |
| Home | -0.58 | 285.00 | -1.60 | -1.30 | 0.13 |
| Money | 0.11 | 285.00 | 0.22 | -0.85 | 1.07 |
| Religion | 2.80 | 285.00 | 1.81 | -0.24 | 5.84 |
| Death | 1.69 | 285.00 | 0.92 | -1.93 | 5.31 |
| Informal | -0.03 | 285.00 | -1.11 | -0.08 | 0.02 |
| Swear | 2.13 | 285.00 | 0.60 | -4.87 | 9.14 |
| Assent | -0.03 | 285.00 | -0.93 | -0.10 | 0.04 |
| Non-fluences | -0.06 | 285.00 | -1.11 | -0.18 | 0.05 |
| Filler words | -0.01 | 285.00 | -0.11 | -0.17 | 0.16 |
| Language Style Matching* | -0.69 | 285 | -0.75 | -2.49 | 1.10 |
| *Note.* Each row represents the quadratic relationship between resident bias and the specified LIWC language variable. * indicates a variable examined in the initial analysis. | | | | | |

| **Table D.** Moderating Effects of Implicit Bias on Language-Patient Satisfaction Relationship | | | | | |
| --- | --- | --- | --- | --- | --- |
| Language Variable | b | df | t | 95% CI | |
| Function words | -0.14 | 283.00 | -1.78 | -0.30 | 0.01 |
| Pronouns | -0.22 | 283.00 | -1.93 | -0.45 | 0.00 |
| Personal pronouns | -0.12 | 283.00 | -1.15 | -0.33 | 0.08 |
| 1^st^ person singular pronouns* | -0.46 | 283.00 | -2.56 | -0.81 | -0.11 |
| 1^st^ person plural pronouns | -0.02 | 283.00 | -0.06 | -0.50 | 0.47 |
| 2^nd^ person pronouns | 0.13 | 283.00 | 1.04 | -0.11 | 0.37 |
| 3^rd^ person singular pronouns | -0.36 | 283.00 | -1.09 | -1.00 | 0.27 |
| 3^rd^ person plural pronouns | -0.33 | 283.00 | -0.75 | -1.20 | 0.53 |
| Impersonal pronouns | -0.11 | 283.00 | -0.84 | -0.37 | 0.15 |
| Articles | 0.23 | 283.00 | 1.29 | -0.12 | 0.59 |
| Prepositions | -0.08 | 283.00 | -0.52 | -0.39 | 0.23 |
| Auxiliary verbs | -0.40 | 283.00 | -2.36 | -0.73 | -0.07 |
| Common adverbs | -0.06 | 283.00 | -0.34 | -0.42 | 0.30 |
| Conjunctions | -0.01 | 283.00 | -0.04 | -0.28 | 0.27 |
| Negations | -0.80 | 283.00 | -2.28 | -1.48 | -0.12 |
| Common verbs | -0.10 | 283.00 | -0.85 | -0.32 | 0.12 |
| Common adjectives | 0.29 | 283.00 | 1.06 | -0.25 | 0.83 |
| Comparisons | -0.44 | 283.00 | -1.44 | -1.05 | 0.16 |
| Interrogatives | -0.78 | 283.00 | -1.86 | -1.59 | 0.04 |
| Numbers | -0.15 | 283.00 | -0.62 | -0.60 | 0.31 |
| Quantifiers | 0.11 | 283.00 | 0.33 | -0.54 | 0.76 |
| Affective processes | 0.21 | 283.00 | 1.75 | -0.02 | 0.44 |
| Positive emotions | 0.24 | 283.00 | 1.98 | 0.00 | 0.48 |
| Negative emotions | -0.11 | 283.00 | -0.39 | -0.69 | 0.46 |
| Anxiety words* | 0.14 | 283.00 | 0.21 | -1.15 | 1.42 |
| Anger words | 2.04 | 283.00 | 0.97 | -2.05 | 6.14 |
| Sadness words | 0.25 | 283.00 | 0.29 | -1.39 | 1.88 |
| Social Processes | 0.00 | 283.00 | 0.03 | -0.21 | 0.22 |
| Family words | -0.64 | 283.00 | -0.73 | -2.36 | 1.08 |
| Friend | 4.19 | 283.00 | 2.30 | 0.64 | 7.75 |
| Female references | -0.66 | 283.00 | -1.66 | -1.44 | 0.12 |
| Male references | -0.69 | 283.00 | -1.16 | -1.84 | 0.47 |
| Cognitive processes | -0.29 | 283.00 | -2.95 | -0.49 | -0.10 |
| Insight words | -0.67 | 283.00 | -2.15 | -1.28 | -0.06 |
| Causation words | 0.07 | 283.00 | 0.18 | -0.75 | 0.90 |
| Discrepancy words | -0.85 | 283.00 | -2.76 | -1.45 | -0.25 |
| Tentative words | -0.18 | 283.00 | -1.02 | -0.53 | 0.17 |
| *Note.* Each row represents the interaction effect of resident bias and the specified LIWC language variable. * indicates a variable examined in the initial analysis. | | | | | |
| **Table D, continued** | | | | | |
| Language Variable | b | df | t | 95% CI | |
| Certainty words | -0.20 | 283.00 | -0.64 | -0.82 | 0.42 |
| Differentiation | -0.65 | 283.00 | -3.18 | -1.04 | -0.25 |
| Perceptual processes | 0.27 | 283.00 | 1.30 | -0.13 | 0.67 |
| See | 0.30 | 283.00 | 0.99 | -0.30 | 0.90 |
| Hear | -0.10 | 283.00 | -0.14 | -1.45 | 1.26 |
| Feel | 0.18 | 283.00 | 0.52 | -0.48 | 0.83 |
| Biological | -0.11 | 283.00 | -0.82 | -0.38 | 0.16 |
| Body | 0.07 | 283.00 | 0.24 | -0.47 | 0.61 |
| Health | -0.14 | 283.00 | -0.68 | -0.54 | 0.26 |
| Sexual | -2.14 | 283.00 | -2.40 | -3.86 | -0.40 |
| Ingestion | -0.33 | 283.00 | -1.03 | -0.94 | 0.29 |
| Drives | 0.29 | 283.00 | 1.92 | 0.00 | 0.59 |
| Affiliation | 0.17 | 283.00 | 0.75 | -0.27 | 0.60 |
| Achievement | 0.06 | 283.00 | 0.14 | -0.84 | 0.97 |
| Power | -0.19 | 283.00 | -0.48 | -0.98 | 0.59 |
| Reward | 0.63 | 283.00 | 2.41 | 0.12 | 1.13 |
| Risk | -0.34 | 283.00 | -0.42 | -1.89 | 1.22 |
| Past-focused | -0.32 | 283.00 | -1.36 | -0.79 | 0.14 |
| Present-focused | -0.03 | 283.00 | -0.23 | -0.26 | 0.20 |
| Future-focused* | 0.44 | 283.00 | 1.74 | -0.05 | 0.93 |
| Relativity (motion, space, time)* | 0.06 | 283.00 | 0.54 | -0.16 | 0.27 |
| Motion | 0.01 | 283.00 | 0.04 | -0.60 | 0.63 |
| Space | -0.13 | 283.00 | -0.82 | -0.44 | 0.18 |
| Time* | 0.23 | 283.00 | 1.46 | -0.08 | 0.54 |
| Work* | -0.52 | 283.00 | -1.36 | -1.27 | 0.23 |
| Leisure | 0.96 | 283.00 | 1.57 | -0.24 | 2.16 |
| Home | -0.21 | 283.00 | -0.17 | -2.67 | 2.26 |
| Money | 2.12 | 283.00 | 1.39 | -0.86 | 5.20 |
| Religion | 3.19 | 283.00 | 0.59 | -7.31 | 13.70 |
| Death | 8.25 | 283.00 | 1.15 | -5.78 | 22.27 |
| Informal | 0.19 | 283.00 | 2.64 | 0.05 | 0.33 |
| Swear | -8.58 | 283.00 | -0.76 | -30.66 | 13.50 |
| Assent | 0.24 | 283.00 | 2.53 | 0.06 | 0.43 |
| Non-fluences | 0.28 | 283.00 | 1.69 | -0.04 | 0.58 |
| Filler words | 0.01 | 283.00 | 0.07 | -0.41 | 0.44 |
| Language Style Matching* | -5.71 | 283.00 | -2.34 | -10.47 | -0.94 |
| *Note.* Each row represents the interaction effect of resident bias and the specified LIWC language variable. * indicates a variable examined in the initial analysis. | | | | | |
