# Supplementary material for: Examining the relationships among physician implicit bias, language, and Hispanic patient satisfaction
Source: PLoS One. 2025 Dec 31;20(12):e0338699. doi: 10.1371/journal.pone.0338699 (PMC12755806; doi:10.1371/journal.pone.0338699)
Supplement: S1 Appendix — (DOCX) [file pone.0338699.s002.docx]

**Supplementary Appendix A**

**Resident Language & Patient Satisfaction**

Before applying corrections for Type 1 error inflation, patients whose physicians used a larger number of articles, *b* = 0.21, *t*(285) = 3.04, *95% CI:* [0.07, 0.34], used more first-person plural pronouns (e.g. “we”), *b* = 0.20, *t*(285) = 2.14, *95% CI:* [0.02, 0.39], used more drives-related language, *b* = 0.14, *t*(285) = 2.60, *95% CI:* [0.04, 0.25], and used more affiliation-related language, *b* = 0.19, *t*(285) = 2.16, *95% CI:* [0.02, 0.35], reported higher satisfaction with their visit. Patients whose physicians used more perceptual language reported lower patient satisfaction, b = -0.16, t(285) = -2.04, 95% CI: [-0.31, -0.01]. Given the large number (75) of hypotheses tested, we controlled for the false discovery rate (Benjamini & Hochberg, 1995). This approach is an alternative to family wise error corrections such as Bonferroni corrections and results in higher statistical power. After these corrections, only the effect of use of articles on patient satisfaction remained significant.

**Moderating Effects of Implicit bias on Language-Patient Satisfaction Relationships**

There were 3 language variables that yielded significant effects only for high implicit bias physicians– cognitive process words, interaction: *b* = -0.28, *t*(283) = -2.95, *95% CI:* [-0.49, -0.10], discrepancy words, interaction *b* = -0.85, *t*(283) = -2.76, *95% CI:* [-1.45, -0.25], and reward-related words, interaction *b* = 0.63, *t*(283) = 2.41, *95% CI:* [0.12, 1.13]. For only high bias physicians, greater use of reward-related words was associated with greater patient satisfaction. In contrast, greater use of cognitive process and discrepancy words by high bias physicians was associated with less patient satisfaction.

Among physicians with low levels of implicit bias, the relationship between language use and patient satisfaction was significant for 6 language variables. For physicians with lower levels of implicit bias, greater use of auxiliary verbs, interaction *b* = -0.40, *t*(283) = -2.36, *95% CI:* [-0.73, -0.07], negations, interaction *b* = -0.80, *t*(283) = -2.28, *95% CI:* [-1.48, -0.12] and insight-related words, interaction *b* = -0.67, *t*(283) = -2.15, *95% CI*: -1.28, -0.06], was associated with greater levels of patient satisfaction. For physicians with average or high levels of implicit bias, these language variables were unrelated to patient satisfaction. In contrast, when physicians with lower levels of implicit bias used more informal language, interaction b = 0.19, t(283) = 2.64, 95% CI: [0.05, 0.33], assent words, interaction b = 0.24, t(283) = 2.53, 95% CI: [0.06, 0.43], and friend-related words, interaction b = 4.19, t(283) = 2.30, 95% CI: [0.64, 7.75], patients reported lower levels of satisfaction. For these language variables there was no relationship between language use and patient satisfaction at average or high levels of implicit bias.

One moderating relationship involving differentiation language, displayed a crossover interaction, *b* = -0.65, *t*(283) = -3.18, *95% CI*: [-1.04, -0.25]. For low implicit bias physicians, greater use of differentiation language was associated with greater patient satisfaction. This effect was attenuated at mean levels of implicit bias. For physicians with high levels of implicit bias, greater use of differentiation language was associated with less patient satisfaction.

These findings should be interpreted with caution. After controlling for the false discovery rate, no interaction effect met the corrected criterion for statistical significance.
